# Supplementary material for: Are carbon emissions trading and green financial instruments synergistic? -Comprehensive quantitative research based on content analysis
Source: PLoS One. 2024 Mar 7;19(3):e0298601. doi: 10.1371/journal.pone.0298601 (PMC10919601; doi:10.1371/journal.pone.0298601)
Supplement: S1 Data — (ZIP) [file pone.0298601.s001.zip › Data/地级市Z_F工作报告.zip!/║╙─╧/║╫▒┌╩╨/└┤╫╘╣1⁄2╓┌║┼í╛┬φ┐╦╩2╛▌═°í┐_2006.docx]

各位代表：

　　现在，我代表市人民政府，向大会作政府工作报告，请予审议，并请各位政协委员提出意见。

　　一、2005年及“十五”以来工作回顾

　　2005年，在省委、省政府和市委的正确领导下，市政府团结带领全市人民，认真实践“三个代表”重要思想，全面落实科学发展观，求真务实，扎实工作，圆满完成了市八届人大二次会议确定的各项目标任务。

国民经济发展势头强劲。按新基数、新办法初步核算，全市生产总值完成186.05亿元，同比增长15.8%，居全省第3位，是10年来最高增速(按老基数、老办法测算，完成193亿元，增长16%)。财政一般预算收入突破8亿元，增长43.9%，居全省第3位，是20年来最高增速；一般预算支出18.2亿元，增长45.1%，居全省首位。全社会固定资产投资达到75.5亿元，增长54.3%，是10年来最高增速，其中城镇投资66.5亿元，增长55.2%，居全省第4位。规模以上工业增加值83.98亿元，增长25%，居全省第4位，比上年前移9个位次；实现利润7.04亿元，增长45.5%；综合经济效益指数达到159.5%，提高28.1个百分点。金融机构各项存款余额比年初增加18.3亿元，增长16.48%，居全省第6位；各项贷款余额比年初增加19.5亿元，增长21%，居全省第2位，金融资产质量实现根本好转，信用环境和投资环境得到明显改善。经济的快速发展，使人民群众得到了更多的实惠。城镇居民人均可支配收入、农民人均纯收入分别达到7898元和3469元，增长16.3%和15.1%，增速分别居全省第1位、第2位。社会消费品零售总额40.4亿元，增长14.7%，居全省第2位。粮食总产92.91万吨，单产402公斤，均创历史新高。县区经济呈现竞相发展的强劲势头，淇县、浚县生产总值分别增长20.1%和15%，淇县跨入财政一般预算收入超亿元县行列，淇滨区、鹤山区、山城区财政一般预算收入增速分别居全省区级第1、第2和第4位。招商引资和大项目建设实现新突破。全市到位市外直接投资达到66亿元，增长30%。引进超亿元项目19个，总投资规模47亿元。合同利用境外直接投资8211万美元，增长105%，居全省第3位；实际利用境外直接投资3023万美元，增长115%，居全省第2位。无人驾驶飞机、全自动无油烟炒菜机、汽车模具、移动通信接收发射装置、人工晶体、生物发电等一批高新技术项目和循环经济项目已经投产或正在建设。“全国民营企业家看鹤壁”活动全方位展示了我市的新形象，得到省委、省政府的充分肯定，在全国民营企业界产生了积极反响。在招商引资的强力带动下，大项目建设取得新突破。全市在建超5000万元项目65个，总投资规模170亿元，其中超亿元项目29个，总投资150亿元。项目单体规模和质量明显提高。电厂二期1号、2号机组双双投入运行，电厂三期、热电联供项目进入施工高潮，投资2.68亿元的生物发电项目开工建设。水泥厂三期建成投产，新型干法水泥熟料日生产能力达到1万吨，成为全省最大的单体水泥生产基地。成功实施总投资7.8亿元的鹤煤集团与泰国万浦集团合作改扩建项目。投资20亿元的年产60万吨甲醇项目正在抓紧进行开工前的各项工作。采煤沉陷治理项目进展顺利，第一批4个住宅小区正在抓紧建设。盘石头水库主体工程完工。新老区快速通道基本完工。调整产业结构和转变经济增长方式迈出坚实步伐。成立了循环经济、煤化工、汽车零部件等重点产业推进工作小组，加快重点产业推进步伐。被确定为全省煤化工、汽车零部件、食品工业、纺织服装产业基地和高新技术产业集群之一。煤电、建材、金属镁、畜产品加工等循环产业链进一步拓展延伸，被确定为全国首批7个循环经济试点市之一。经过积极争取，被列为全国11个建筑节能示范城市之一，5年内将得到国家1.5亿元建筑节能改造资金支持。全省循环经济发展论坛、循环经济研讨暨经验交流会先后在我市召开。“食品安全关键技术应用示范区”顺利通过国家验收。畜牧业产值达到35亿元，加工产值41亿元，分别增长10.4%和32.3%；大用、永达公司双双跻身“中国肉类食品50强”，成功创获“中国名牌产品”，大用公司成为首批“国家认定企业技术中心”，科技部将重点支持其开展自主研发活动，承担科研计划。工业集中区建设取得重要进展，新入驻企业63家，总投资21亿元，进展明显加快。市财政用于支持重点企业改制改造和结构调整的投入近5000万元。房地产、商业和市场建设的规模和档次得到进一步提升，建筑业、房地产业开始成为拉动经济发展、增加财政收入新的支柱产业。实现旅游直接收入5342.6万元，增长53.3%。豫北粮食仓储物流交易中心等项目已经建成或正在建设之中。积极推进金属镁污染企业专项治理工作。在关闭51家污染严重的小金属镁企业的同时，注重引进和支持上规模、上档次的金属镁企业，努力促进产业优化升级，使我市金属镁产业展现出健康发展的前景。按照要求对23家小煤矿实施了关闭。畜牧养殖业面源污染治理全面展开。山城污水处理厂部分通过验收，淇滨污水处理厂投入试运行，市垃圾处理场和淇县、浚县污水处理厂正在建设。全市工业污染源达标排放率达到95%以上。城市饮用水源地水质达标率保持在100%，出境河流水质明显改善，市区空气质量达到二级以上天数301天。大规模开展淇河两岸、快速通道两侧、新老区连接带以及太行山区生态绿化，完成造林绿化10.2万亩，淇县平原绿化高级达标顺利通过省级验收。城镇化进程进一步加快。按照“以新区为中心，以老区、浚县和淇县为三个支撑点，突出抓好产业支撑、完善功能、绿化美化、提高管理水平等重点工作，全方位加快城镇化步伐”的整体思路，高度重视并积极推进城建工作。编制完善了市区和两县县城总体规划，努力抓好规划审批和批后管理，规划的引导调控作用得到进一步发挥。与规划紧密结合，进一步严格土地管理，做好土地资源策划运作，收到了明显效益。新区建成区面积达到17.2平方公里，新增与改造公共绿地49.3万平方米，绿化覆盖率达到40%，顺利通过中国人居环境范例奖省级验收。一批教育、文化、卫生等公共服务设施投入使用，城市功能进一步完善。城中村改造取得重要进展。老区整治改造建设迈出积极步伐，城市环境得到初步改善。两县县城改造、扩张步伐加快，城市经济实力和人口吸纳能力得到增强。小城镇建设也取得积极进展。全市城镇化开始进入加快发展的新阶段。成功举办了河南鹤壁城乡一体化高层咨询论证会，城乡一体化工作正在积极探索与推进之中。全市城镇化率超过42%，被省确定为城乡一体化试点市。改革向深度推进向广度拓展。国有企业改制基本完成，企业活力和市场竞争力明显增强。国有粮食购销企业减员分流和产权制度改革提前顺利完成，实现盈利首次突破400万元。农村信用社改革进展顺利，经济效益整体好转。政府投资项目代建制进一步完善，已代建项目13个，总投资规模近亿元，项目建设规范管理水平明显提高。乡镇机构改革提前完成，实现了分流人员的平稳离岗，乡镇机构、领导职数分别精简40%和32%。干线公路养护、交通规费征收、道路运输场站管理体制改革平稳顺利完成。市政公用事业、勘察设计业、国有建筑业改革取得较大进展。非公有制经济快速健康发展，个体工商户、私营企业分别比年初增长23.9%、21.5%，增速居全省第2位和第7位。非公有制经济增加值占全市生产总值的比重达到51%，提高4.7个百分点。非公有制企业吸纳劳动力15.2万人，占全市从业人员的55%。和谐社会建设全面扎实推进。市委、市政府承诺的与群众生活密切相关的实事，当年任务已圆满或超额完成。下岗失业人员再就业技能培训、农民工技能培训分别达到9949人和7003人，完成年目标的153%和117%。被确定为全国创业培训重点联系城市。城镇新增就业3.87万人，下岗失业人员再就业1.72万人，均完成省定目标的2.3倍以上，城镇新增就业人员完成比例居全省第1位，城镇无业人员比上年减少0.78万人，降幅达36.6%。劳务输出18.4万人次。解决了城镇各类灵活就业人员参保难问题，社会保险覆盖面进一步扩大。城乡困难群众救助体系进一步完善。特困企业退休人员大病救助工作取得初步成效。为6万多名城市低保对象发放低保金3620万元。建立了农村低保制度，3.1万名农村特困群众纳入了低保。3个城区均被列为全省城市医疗救助工作试点，农村医疗救助工作也取得积极进展。慈善工作扎实推进。发放农村义务教育阶段贫困家庭学生“两免一补”资金806万元，补助73830人次。儿童福利院、老年康复中心、救助管理站建设完成开工前的各项准备工作。投入2.47亿元大力改善农村生产生活条件，比上年增加6063万元，是历年投入增加最多的一年。投资1.33亿元新建改建县乡村道路396公里。投资2489万元解决了卫河、共渠沿岸69个行政村11万群众饮水安全问题。基础教育进一步得到巩固，高中教育、职业教育、高等教育发展步伐加快。高中阶段在校学生46136人，比上年增加3977人，增长9.4%。农村中小学现代远程教育试点工程圆满完成。新课程教学改革取得良好效果。新区新建的4所学校顺利实现招生。教育资源整合工作稳步推进，撤校49所、并点47个。农村中小学危房改造提前一年完成省定目标任务。玉米亩产创黄淮海夏玉米最高，“浚单18玉米杂交种的示范与推广”荣获2005年度河南省科技进步一等奖。鹤煤集团、国营浚县农场等企业所属学校移交地方管理工作顺利完成。“三馆合一”的市文化中心对公众开放取得良好社会效果。文化产业发展取得积极进展。浚县石雕城被确定为全省文化产业示范基地。在全省率先完成了广电网络整合，被列为全省首家数字电视整体平移试点市和户外电视大屏幕、移动电视城市，广播电视信息化、现代化水平进一步提升。顺利完成7个乡镇卫生院和“120”紧急救援指挥中心建设，公共卫生体系和农村卫生服务能力得到进一步加强。浚县新型农村合作医疗试点工作走在全省前列。在全省首创政府药品集中采购新模式，成效明显。全年未发生一起食品卫生事件，是全省唯一没有发生食品卫生事件的省辖市。积极承办高水平体育赛事，开放和增加小型便民体育设施，群众体育生活更加丰富。代表国家参加第31届大金属球世界地掷球锦标赛进入8强，参加第9届小金属球亚洲地掷球锦标赛获得亚军。残疾人康复、就业、扶贫救助等工作取得新成效。残疾人就业基金征缴取得积极进展。人口和计划生育工作成效显著，浚县被评为国家级优质服务、药具管理、协会工作先进县，淇县、鹤山区被评为国家级行业作风建设、协会工作和宣传教育工作先进县区。开展了以“一抓四进”为载体的“法制宣传教育年”活动，取得明显成效。坚持向人大报告工作、向政协通报情况，认真办理人大代表建议和政协提案269件。“双创”工作扎实推进。第五届村委会换届工作顺利完成。再次被评为“双拥模范城”。社会治安形势进一步好转，刑事、治安案件同比分别下降32.6%和45%，命案起数降至16年来最低，现行命案和命案积案破案率继续双双保持全省第一，群众对社会治安的满意率达到93.7%，被评为全省社会治安综合治理先进市。安全生产事故起数下降7.6%，死亡人数比上年下降39.8%，安全生产管理成效显著。依法做好信访工作，进一步健全应急机制，继续保持了在全省的先进位次。发展环境进一步优化。努力推进政府部门队伍建设和效能建设。进一步改善服务态度，提高办事效率，强化效能监察。大力倡导调研学习和提合理化建议的风气，进一步优化工作流程和推行精细化管理。实行招商引资和重点建设项目代办督办等新机制，工作流程特别是建设领域项目审批流程不断优化，窗口服务质量明显提升。实行重点工作台帐制。充分运用电子政务促进工作效率、质量和公务员素质的提高。创新完善了一系列制度规定，更加切实地落实了收支两条线政策，更加严格规范了对企业的各种检查、收费和罚款行为，从严查处破坏环境的典型案件，使经济发展环境得到进一步优化。

　　2005年全市经济社会发展主要指标均进入全省前4位，保持了既快又好的良好势头，40多个部门的工作进入全省先进行列，121项工作受到国家、省委、省政府和省直部门的表彰，体现了全市工作的特色、优质和全方位的协调推进。全市生产总值占全省的比重由“九五”末的1.66%提高到1.77%，“十五”期间年均增长13.9%，高出“九五”5个百分点，高于全省平均水平2.5个百分点;人均生产总值2004年突破10000元，2005年接近13000元，居全省第7位，比“九五”末增长1.1倍。综合经济效益指数由72.8%提高到159.5%，在全省前移了4个位次。财政一般预算收入比“九五”末增长近2倍，年均增长23.9%，高于“九五”10.4个百分点。全社会固定资产投资累计完成203.6亿元，是“九五”时期的2.5倍。城镇居民人均可支配收入在全省前移6个位次，年均增长14.9%，高于“九五”10.7个百分点。农民人均纯收入已经超过全国平均水平，在全省前移4个位次，年均增长10.5%。投入更多的精力和财力解决关系群众切身利益的问题，财政对教科文卫等社会事业的投入年均增长21.1%，高于“九五”11.8个百分点。可以说，新世纪的头五年，是我市经济总量连续迈上新台阶、综合实力实现新跨越的五年，是各项改革不断深化、对外开放取得历史性突破的五年，是城乡面貌显著改善、城市形象和品位提升最明显的五年，是经济社会协调健康发展、人民群众得到实惠更多的五年，是各项工作在全省出特色、出亮点的五年。我市经济社会发展迈上了新的战略起点，进入了全新时期。更为可贵的是，全市广大干部群众在积极探索和努力实践中，展现出改革开放、创新拼搏、求真务实的良好风貌，形成了齐心协力谋发展、和衷共济创大业的浓厚氛围。一个生机勃勃、奋发向上的新鹤壁，展现在世人面前。

　　各位代表！以上成绩的取得，是省委、省政府和市委正确领导的结果，是市人大、市政协大力支持的结果，是全市人民努力奋斗的结果。在此，我代表市人民政府，向全市广大工人、农民、知识分子、干部、驻鹤部队指战员、武警官兵、公安民警以及社会各界人士致以崇高的敬意！向关心、支持我市现代化建设的朋友们表示衷心的感谢！

　　我们也清醒地看到，我市经济社会发展任务繁重、困难较多，工作中还有不少需要改进和加强的地方。产业结构、规模需要加快优化、扩张的步伐，第三产业的发展需要进一步加快。财源建设需要继续加强。城镇居民收入与消费仍处在较低水平，少数群众的生活还相当困难。农村社会事业发展相对滞后。一些政府工作人员的责任意识、服务意识、工作作风、办事效率和工作质量以及创新能力，与新时期的要求相比还有不小差距。经济发展环境还需要进一步优化。对此,我们一定高度重视,以更大的努力、更有效的措施加以解决。

　　二、“十一五”发展的基本思路与重点

　　“十一五”时期，是我市全面建设小康社会、奋力实现“两个率先”的关键时期，也是必须紧紧抓住并且可以大有作为的战略机遇期。从外部大的环境看，我国仍将处于经济快速增长的阶段，国内外产业结构调整和转移加快，国家对中部地区支持力度进一步加大，都为我市保持经济社会快速发展提供了良好的外部环境。从我市自身情况看，“十五”期间综合经济实力的大幅提升，为“十一五”时期经济加快发展积蓄了能量、打下了基础。我市成为国家循环经济试点市和省城乡一体化试点市，以及全省规划的煤化工、食品工业、汽车零部件、纺织服装产业基地和高新技术产业集群之一，产业发展方向进一步明确，为全市“十一五”时期的发展提供了难得的动力和机遇。同时也要看到，全国全省各地你追我赶、竞相发展，容不得我们有任何自满和松懈。我们必须审时度势，树立小市要有大作为、小市能有大作为的目标和信心，以只争朝夕的精神，以务实苦干的态度，把各项工作做得更加主动，更有创新，更富成效，确保“十一五”全市经济社会发展取得更加辉煌的成绩。

　　“十一五”时期我市经济社会发展的总体要求是：坚持以科学发展观统领经济社会发展全局，进一步加快工业化、城镇化和农业现代化进程。通过大力调整结构、转变增长方式，努力提高经济增长的质量效益和区域竞争力。坚持不懈地抓好招商引资和大项目建设，大力发展循环经济，加快构建节约型社会，积极推进城乡一体化。壮大优势产业，做强特色产业，建设特色城市。力争支柱产业省内靠前，特色产业国内有名，经济总量快速翻番，人均指标全省领先，构建“富裕、文明、和谐、生态”的新鹤壁，努力为中原崛起做出更大贡献。

主要预期目标是：全市生产总值年均增长14%以上，2010年力争比“十五”末翻一番；人均生产总值达到3000美元。财政一般预算收入年均增长16%以上，2010年比“十五”末翻一番。全社会固定资产投资累计达到500亿元以上。顺利完成“十一五”目标任务，实现鹤壁发展的新跨越，必须重点抓好以下六个方面的工作：一是继续实施开放带动战略，着力推进招商引资和大项目建设。紧紧围绕我市业已明确的重点产业，在做大做强重点企业和构建产业集群上下功夫，在引进高新技术和精深加工项目上下功夫，在引进国内外知名企业上下功夫，在引进和推进大项目建设上下功夫。“十一五”期间，全市招商引资累计到位资金力争达到400亿元以上。二是大力发展循环经济，走资源节约型发展道路。以煤炭、电力、建材、金属镁、畜牧加工为主体，加快构建比较完善的工业、农业循环经济体系，提高资源的综合利用率，降低单位产值能源消耗水平，走资源节约型、环境友好型的发展路子。2010年单位生产总值能耗比“十五”末降低22%以上。三是坚持以新区为龙头，进一步加快城镇化进程。以强化产业支撑为根本，通过增强城市功能，完善政策体系，突出强化新区的人口聚集能力，同时充分发挥山城区和浚县、淇县以及重点镇的人口吸纳作用，努力加快我市农村人口向城镇转移的步伐。“十一五”末，全市城镇化率力争达到55%。四是认真抓好社会主义新农村建设，积极推进城乡一体化工作。准确把握建设社会主义新农村的内涵，科学制定规划，明确工作重点，加强示范带动，着力解决农民最关心、最迫切需要解决的问题，努力改善农村生产生活条件，促进农村整体面貌有明显改观，促进城乡社会协调发展。五是坚持改革创新，为经济社会快速协调发展提供体制条件。继续深化行政管理体制改革、国有企业改革和农村综合改革。积极推进文化、旅游、财税、社会事业、市政公用行业等领域的改革。积极引导、大力支持非公有制经济发展，提升非公有制经济在全市发展中的地位和作用。六是坚持以人为本，加快构建和谐社会。全面发展各项社会事业，解决好就业再就业、社会保障等人民群众最为关心的问题。加强社会治安综合治理，切实做好信访稳定和安全生产工作。加强精神文明和政治文明建设，巩固发展民主团结、生动活泼、安定和谐的社会政治局面。

　　《鹤壁市国民经济和社会发展第十一个五年规划纲要(草案)》及说明已印发各位代表，请予审议。

　　三、2006年经济社会发展主要目标和任务

　　2006年是“十一五”规划开局之年。经济社会发展的主要预期目标是：全市生产总值增长15%；全社会固定资产投资增长25%以上，超过100亿元；规模以上工业增加值增长20%，超过100亿元；招商引资到位市外直接投资超过70亿元，力争80亿元；实际利用境外直接投资确保3600万美元，力争超过5000万美元；社会消费品零售总额增长15%；财政一般预算收入同比增长16%；城镇居民人均可支配收入增长12%，农民人均纯收入增长9%；单位生产总值能耗降低5.5%左右；人口自然增长率控制在5‰以内；城镇新增就业超过3万人。

(一)继续强力推进招商引资和大项目建设，进一步提升整体质量和水平

　　坚持把对外开放作为我市经济社会发展的主战略，围绕国家、省宏观政策和产业方向，围绕我市重点工作、产业布局开展招商，增强招商引资的针对性。重视引进技术含量高的项目，对就业和税收贡献大的项目，有利于我市重点产业发展的项目，使更多的引进项目上规模、上水平、出效益。巩固拓展招商基地建设成果，增加招商引资项目储备。与我市产业发展重点相结合，谋划举办金属镁、煤化工、汽车零部件等行业发展高层论坛，推动招商引资工作。加强招商引资项目的综合分析，全面掌握招商引资的实际效果，促进招商引资质量、效益的不断提高。大力引进境外资金，认真做好第四届中国河南国际投资贸易洽谈会等参展工作，力求取得积极成效。各县区、市直主要招商引资部门要力争各引进1个投资规模超5亿元的项目,或世界500强、国内500强企业投资的超亿元项目，并为引进2个以上投资规模超10亿元的项目而共同努力。进一步加大专业市场、现代物流、教育、文化、卫生、旅游、休闲度假、公用事业等领域的招商引资力度。加快推进工业集中区基础设施和服务体系建设，增强项目承接和配套服务能力。引导帮助企业进一步提高自主招商的意识，增强以商招商的成效。有针对性地推动行业协会、商会等中介组织建设，搭建政府与企业沟通的桥梁。完善落实代办督办等各项制度和服务措施，进一步提高行政服务中心窗口工作效率和服务质量，抓好对落地项目的跟踪服务，使企业早日建成、早见效益。进一步明确县区、部门责任，切实解决好建设项目及企业的施工和经营环境问题。做好出口型企业政策、信息、环境协调等服务，进一步扩大出口规模，优化出口结构。

　　积极推进和服务好大项目建设。切实抓好项目核准、资金落实、征地拆迁、建设进度等方面的工作，确保大项目建设高速度、高质量推进，尽快投产见效。热电联供项目确保按计划建成投产,实现向新区供热。电厂三期尽早进入全面施工高潮。新老区快速通道确保6月底前竣工通车。积极做好长治—鹤壁—泰安铁路、山西—鹤壁高速公路前期工作,争取早日开工。认真做好南水北调中线工程、郑州—石家庄铁路客运专线、京珠高速公路鹤壁段拓宽改造、107国道鹤壁段改建升级等工程开工前后的各项配合服务。扎实推进采煤沉陷治理项目。积极做好盘石头水库移民接收工作，加快库区生态保护和综合开发利用，使水库早日下闸蓄水、发挥效益。

(二)加快产业结构优化升级步伐，努力构建优势产业集群

　　依托新区大力推进高新技术企业密集区建设，着力发展电子信息、新型材料、生物制药等产业，构建高新技术产业集群，使之成规模，树品牌，在我市优化产业结构、转变经济增长方式过程中发挥先导作用。按照“十一五”全省重点产业规划布局，大力发展煤化工、建材、金属镁冶炼及精深加工、汽车零部件、食品加工、纺织服装等带动性强、关联度高、对长远发展有重要影响的产业，实现全市产业结构的优化升级。协调支持鹤煤集团加快煤矿改扩建和新建步伐，积极推进煤化工及后续加工项目，抓紧开工年产60万吨甲醇项目。加快电厂三期、热电联供等电源项目建设，着手开展电厂四期可行性研究等前期工作。积极谋划新型干法水泥产能进一步扩张，努力发展建筑陶瓷。加快煤矸石和粉煤灰烧结砖等项目建设，积极引进节能型建材企业。加快推进金属镁冶炼和精深加工系列项目，争取我市金属镁产业发展取得重大进展，向打造全国重要的金属镁精深加工产业基地目标迈进。重点培育发展汽车插接件、模具、轮胎、野战通信车等系列产品，注重新产品、总成产品的引进，加快汽车零部件企业聚集。鼓励现有食品加工企业加快规模扩张，延长加工链条，拓展加工领域，努力实现与国内外有实力企业合资合作的新突破。进一步培育壮大新的龙头加工企业。加强食品安全和防控体系建设。力争全年实现畜牧业产值40亿元、加工产值50亿元。抓住我国发达地区向内地转移劳动密集型产业的机遇，在积极支持现有纺织服装企业加快发展的同时，下力气再引进一批纺织服装企业，壮大产业集群，形成产业优势。进一步整合现有物流资源，积极发展第三方物流，开工建设市货运配载中心，努力引进市外有实力的物流企业。积极策划引进外向型专业物流市场，力争取得突破。

　　进一步谋划、明确各工业集中区产业发展指向，努力吸引同类企业、上下游及横向关联企业向工业区集中，形成有特色的产业集群，建设特色工业园区。认真做好对包括招商引资落地项目在内的市内企业的服务，选择一批具有较大发展潜力的企业给予重点引导、扶持，促其通过多种途径实现再投资、再合作、再发展。引导重点企业加大研发投入，积极申报省级、国家级技术研发中心，提高自主创新能力。引导支持有条件的企业，积极争创省、国家名牌产品和著名、驰名商标。加强企业家队伍建设，培养一批有积极进取心、有现代企业运作能力、有社会责任感的企业家。

　　抓紧谋划制定促进我市服务业发展的措施办法，力争取得较大进展，努力改变我市服务业发展相对滞后的局面，实现三次产业协调发展。以新区为中心，以“一河两城三山”为依托，加强旅游资源整合和联合宣传推介，加快景区改制和招商引资步伐，更好地发挥中国优秀旅游城市的品牌效应。结合新区扩张与老区改造，提升我市房地产、餐饮、商业等传统服务业档次。大力推进文化产业发展，积极谋划推动古陶、石雕、泥塑、剪纸、根雕、演艺、会展等文化产业的扩张，建设文化产品综合展示区和旅游产品市场，努力打造文化精品。结合发展旅游业、完善新区功能，积极发展外向型休闲娱乐业。

(三)大力发展循环经济，加快建设节约型社会

　　把循环经济向更深更广的层次推进，加速构建比较完整的循环经济体系；结合循环经济工作，以节能节水节地节材和资源综合利用为重点，加快节约型社会建设，继续走在全省全国的先进行列。

　　积极开展循环经济试点各项工作，加大相关项目推进力度。引导鼓励企业做好循环经济先进技术的开发、引进和推广工作。支持推动鹤煤集团、万和公司等耗能大户做好节能降耗工作。加快鹤煤集团矿井水综合利用、煤矸石烧结砖、煤层气发电，浚县生物发电等项目建设，努力争取风力发电项目。促进鹤煤集团、华韵公司两个年产2.4亿块煤矸石烧结砖项目建成投产。开工建设鹤煤集团粉煤灰蒸养砖、电厂一期脱硫、同力公司纯低温余热发电等项目。围绕“三大产业基地”和循环产业链，再谋划、建设一批新的循环经济项目，进一步丰富循环经济发展内涵。编制新区北部高新技术企业密集区、牟山工业集中区的生态园区建设规划，努力引进资源综合利用项目，最大限度地发挥资源能源的效益。以村镇和畜牧龙头企业为单位，规划启动3—5个生态农业园区建设。

　　把节约能源资源的理念和要求，贯穿城市规划、建设和管理的各个环节，推广资源节约、替代和循环利用的先进适用技术。切实抓好国家建筑节能示范城市试点工作，全面推进新建建筑节能工作。因地制宜推广浅层地热利用技术。积极引进太阳能利用新技术、新产品。加快发展农业节水灌溉。努力推广多层标准厂房。大力清理整治砖瓦窑厂，在市区范围内禁止使用实心粘土砖，积极推广新型建材，加快节能建筑材料企业建设。完善支持循环经济发展的配套政策措施，建立循环经济和节约型社会统计考核指标体系，纳入政府责任目标管理。大力提倡、率先垂范节俭文明的生活方式，努力营造建设节约型社会的浓厚氛围。

　　认真做好区域内卫河流域水污染综合整治，对重点排污企业实行强制性清洁生产。加强城市特别是老区空气质量监控治理，确保全年空气质量进入全省优良行列。切实加强对非煤矿山开采的监管和整治，严加限制并督促限期做好生态保护和恢复。淇县污水处理厂建成投运，浚县污水处理厂基本完工，山城污水处理厂、淇滨污水处理厂完成管网配套工程并正式运行。市垃圾处理场基本建成并启用。严格按照规定征收污水处理费、垃圾处理费。完善重大污染事件预警应急处置机制，提高应对处置重大污染事件的能力。严防已治理关闭污染企业出现反弹。依法严肃查处环境违法行为。加大淇河绿化投入和保护力度，挖掘发挥人文资源优势，努力打造淇河自然生态文化长廊。组织开展好快速通道两侧、新老区连接带、重点通道、太行山以及淇河公园等重点地带的绿化，浚县实现平原绿化高级达标。

(四)扎实推进城镇化和城乡一体化，努力建设社会主义新农村

　　继续以新区为中心，以老区和两县县城为支撑点，突出以人为本的理念和宜居城市的定位，科学谋划、加快构建独具特色的城市建设新格局。城镇化率达到44%，城镇人口达到65万人左右。重视并做好各项城建规划的编制完善工作，切实抓好规划的实施管理和批后管理，提升城市品位。严格土地管理，进一步做好土地资源开发利用工作，提升土地资源效益，确保土地保值增值和集约节约利用。加快道路、供热、供气、供水、物业、教育、电力、通讯、公交等基础设施和公用事业的建设，努力提高管理和服务水平。新区在规划范围内加快向北产业扩张和向南城区扩张，进一步增强城市功能，完善管理机制，丰富文化内涵，彰显城市特色，进入建设新高潮。续建和新建城市框架范围内主要道路23条段、19公里以上。以道路、供水、供暖等为重点，完善城市基础设施。以突出人性化、提高品位为重点，完善市容市政设施。继续抓好城市绿化，进一步打造精品。加快淇河新区段开发建设。完成二支渠西段和护城河改造，启动二支渠东段建设，建设淇河公园。完善新区供热管网，积极推进新区天然气储气管网建设。全力推动新区规划区内村庄改造，年内力争完成50%的改造任务。积极做好村委会改居委会工作。探索实施城市管理综合执法。依法治理城区马路市场。整顿规范物业市场，进一步提高物业管理水平。统一谋划淇滨区、山城区、鹤山区的经济发展与城镇化布局，增强经济互动和功能对接。老区在加快产业发展的同时，继续完善城市基础设施，提高城市管理水平。结合采煤沉陷治理项目，有计划、有重点地推进城区和城中村改造，努力改善城区面貌和居民生活环境。抓好主干道的改造和净化、绿化、美化，治理占道经营和违章建筑，加强门面房整治。推进居民小区和背街小巷的环境整治。开展城区周边环境治理。探索新老区连接带新型城市建设模式，突出大规模、高品位的生态绿化，实现生态建设和经济开发的良性互动。通过新老区连接带向老区的产业延伸，强化新老区联系与对接。鹤山区着力抓好军鹤线产业带建设和鹤壁集改造建设。淇县加快向北扩张县城、延伸产业，同时做好老城改造，使之与新区发展相呼应。浚县县城在加快向外扩张的同时，积极而科学地推进老城改造，努力展现国家历史文化名城风貌并充分发挥其效应。浚县、淇县县城建成区面积分别达到10平方公里和9平方公里。围绕“人”字形经济带，抓好重点小城镇的功能完善和经济发展。

　　认真落实城乡“六个一体化”五年规划和近期推进城乡一体化工作意见，努力实现重点突破和良好开局。围绕加快城镇化进程，制定完善户籍、土地、教育、医疗卫生、就业、社会保障等配套政策，加快体制创新，抓好试点示范，为城乡交流打开通道，为农村劳动力向城镇转移提供政策平台。完成城乡一体化各项专项规划的修编。完善城乡一体化重点镇、重点村规划并积极组织实施。进一步推动城乡产业布局优化和相互融合。实施城乡公交一体化工程，开工建设16个乡镇客运站，积极推动城市公交向农村延伸。

　　结合推进城镇化和城乡一体化，认真贯彻落实中央、省支农惠农政策措施，进一步做好“三农”工作，在社会主义新农村建设方面取得积极进展。大力扶持农业产业化龙头企业，加快发展壮大步伐，提高农业产业化水平。积极推进农村专业合作经济组织建设，促进农业的规模化、组织化、标准化经营，提高农业、农村和农民的发展能力。继续做好劳务输出工作，引导农村富余劳动力向城镇和非农产业转移，拓宽农民增收渠道。继续对种粮农民进行直接补贴、良种补贴、购置大型农机具补贴。依法保护农民工合法权益，从源头上解决农民工工资拖欠问题。切实加强涉农收费和价格管理，防止农民负担反弹。解决4万农民饮水安全问题。完成236公里“村村通”工程、4万亩中低产田改造工程。积极推广沼气等清洁能源，新增沼气用户2000户。积极推进农村自来水普及、广播电视“村村通”工作。继续做好浚县新型农村合作医疗试点工作，启动淇县农村合作医疗。积极发展农村医疗卫生事业，开展标准化村卫生室试点工作，积极探索县乡村医疗资源纵向合作新途径，切实提高农村医疗服务水平。完善农村文化基础设施。积极谋划、推动农村体育事业发展。继续做好农村贫困家庭学生“两免一补”工作。加大扶贫开发力度，努力使5000名农村贫困人口脱贫。引导帮助重点村抓好道路硬化、绿化、垃圾清运等工作，建立健全村容村貌有效管理机制，形成居住环境良好的新村貌。

(五)积极推进各项改革，为发展提供不竭动力

　　深刻认识加快改革的重要性、紧迫性，坚定不移地推进改革。同时做到改革方案积极稳妥，工作推进深入细致，风险防范及时有效，确保改革平稳顺利进行。

　　工业领域国有企业改革完成最后攻坚，加快推进其他领域国有企业改革。加快行政管理体制改革，全面推进依法行政。进一步改革行政审批体制，理顺政府与企事业单位、中介组织、市场的关系，切实转变政府职能，更好地履行公共管理和社会服务职责，建设责任清晰、运转高效的政府。继续完善政府投资项目代建制，提高代建水平和质量。推进金融生态环境建设，支持帮助各商业银行加强与企业的沟通联系，积极拓展业务。努力拓宽融资渠道，吸引市外金融机构贷款。积极推动城市信用社申报城市商业银行工作。扩大市担保中心资本金规模，进一步发挥担保融资作用。积极推动企业上市和发行企业债券工作。巩固农村税费改革和乡镇机构改革成果，推进农村综合改革。积极推进农村土地流转、农村金融体制、农村流通体制等改革。继续深化财政体制和预算管理制度改革，完善公共财政体系。有步骤地分类推进事业单位改革。加快市政公用行业改革，积极探索污水处理、垃圾处理、城市供热、公交运营等特许经营、市场化运作的路子。推进城市公交车辆公司化运营，加强公交场站设施建设，全面提高城市公交管理服务水平。积极推进文化、旅游、科技、医疗卫生等领域的改革。

(六)着力解决关系群众切身利益的问题，努力建设和谐鹤壁

　　牢记人民政府的基本职责，尽最大努力解决关系群众切身利益的问题，让广大群众充分享受改革发展成果，促进社会和谐、人民安居乐业。

　　努力争取上级资金、集中市县财力，强化工作督促，认真做好市委六届十一次全会确定的与群众生活密切相关的10件实事。

千方百计扩大就业。围绕招商引资和重点产业发展，进一步加大就业再就业工作力度，使之与招商引资、企业发展相互促进，实现就业形势的根本好转。做好1.2万名农村劳动力转移技能培训和8000名失业人员再就业技能培训。落实好小额担保贷款、税费减免等优惠政策，为自谋职业、自主创业创造良好条件。继续做好高校毕业生就业工作。进一步完善社会保障体系。积极做好养老保险费征缴、社会保险扩面工作。确保企业离退休人员养老保险金按时足额发放。提高城市低保标准。全面推进农村低保工作。探索建立失地农民养老保险、医疗保险制度，力争使新区失地农民养老保险参保率达到30%以上，同时积极做好老区失地农民养老保险参保前期工作。扩大农民工参加保险范围，探索农民工大病救助途径。全面推进城乡医疗救助。探索实行困难家庭少年儿童大病救助。完善特困企业退休人员大病保险与救助工作，扩大覆盖面。健全慈善组织网络，努力为困难群众提供更多的救助。加强安全生产和信访稳定工作。进一步提高责任意识和防范意识，以煤矿、非煤矿山、化工企业、道路交通、公共聚集场所和有重大安全隐患的居民区为重点，从源头上防止和杜绝重特大安全事故的发生，努力实现事故起数和死亡人数连续“双降”目标，保持安全生产工作在全省的先进位次。深入宣传贯彻《信访条例》，认真落实信访工作各项制度，进一步加强基层信访工作，积极预防和妥善处置群体性事件，争取重访率明显下降，继续保持信访工作在全省的先进位次，争创全国信访工作先进单位。改进和加强社会治安综合治理，开展平安县区、平安乡镇等创建活动，健全维护社会治安长效机制，确保刑事、治安案件进一步下降，保持良好的社会治安状况，提高人民群众的安全感和满意度，继续争创省社会治安综合治理先进市。全面发展各项社会事业。继续加大教育改革创新力度，全面提升教育教学质量，积极推进素质教育，提高师资特别是农村师资水平。开工建设市特殊教育学校、湘江社区小学、牟山小学。进一步整合教育资源，努力改善薄弱学校办学条件，实现学校平衡协调发展。积极探索推进多元化办学，加快发展民办教育。大力发展职业教育，促进教育发展与产业发展的有效对接。做好职业技术学院迎接全国高职高专人才培养工作水平评估工作。市职业技术学院、技工学校在招商引资合作办学、培养技能型实用人才上取得突破。规范提高幼儿园管理水平。切实保障家庭贫困学生、进城务工农民子女和残疾儿童接受义务教育。注重关键、实用人才引进和创新人才培养，优化科技资源配置，引导企业更加重视新技术、新产品的引进、消化与吸收，提高科技成果转化率，有效发挥科技对经济社会发展的支撑作用。完善市文化中心功能，提升品位，创新管理，并把管理思路、服务体系等延伸到县区。进一步活跃城乡居民特别是农村文化生活，发挥政府和市场的力量，筹划举办一系列有品位、有影响的文化活动，提高演出质量，促进城市文化品位和市民文化修养的提高。加强突发公共卫生事件预防控制和医疗救治体系建设。积极发展社区卫生服务。全面完成乡镇卫生院改造。进一步整顿规范医疗服务秩序，加强医院管理和人才队伍建设。鼓励引导社会资本兴办医疗卫生机构。鼓励支持特色专科医院发展，尽快打出品牌、形成优势。规范完善药品招标采购工作。完善疾病预防控制中心、卫生监督中心功能并尽快发挥作用。探索建立面向中低收入群众的惠民医院。开工建设市体育馆。市体校教学训练楼年内建成并投入使用。积极运作县区体育场所的置换改造与建设。精心规划、建设一批面向群众的小型体育场所和设施。倡导支持以单位、企业、社区为主体开展全民健身活动。加大力度推广地掷球运动，打造“中国地掷球之乡”优势特色体育品牌。繁荣发展新闻出版、广播电视事业。完成部分城区数字电视整体平移，开通户外电视大屏幕和移动电视业务。建成市老干部活动中心。继续做好民族宗教工作。积极做好残疾人工作。严格人口和计划生育政策，稳定低生育水平。 (七)加强精神文明和民主法制建设，全面提高政府整体工作水平

　　切实加强理想信念教育和思想道德建设。实施公民道德建设工程，倡导公民基本道德规范，继续打造诚信鹤壁。加强和改进青少年思想道德建设。深入开展“扫黄打非”。加强社会主义精神文明建设，积极创建省级文明城市。坚持依法治市。更有针对性地开展普法宣传教育，提高公民的法律素质。自觉接受人大及其常委会的法律监督、工作监督和政协的民主监督，严格执行人大及其常委会的决议、决定，支持人大代表依法行使职权，认真办理人大代表建议和政协提案。广泛听取各民主党派、工商联和无党派人士的意见和建议,充分发挥工会、共青团、妇联等人民团体以及协会、商会等社团组织的桥梁纽带作用。自觉接受全市人民的监督。加强法律援助工作,依法保障公民特别是妇女、未成年人和残疾人等弱势群体的合法权益。关心支持驻鹤解放军和武警部队建设。抓好民兵预备役和人防战备建设。认真落实军人优待抚恤和退伍安置政策。深入开展“双拥”活动，增强军政军民团结。

　　突出学习、创新、务实、高效，进一步加强政府自身建设。努力营造浓厚的学习氛围，倡导调查研究和提合理化建议，引导各级政府广大干部职工特别是领导干部，把更多的时间和精力放在对全市经济社会发展的分析思考上，放在对本职工作的不断改进上，增强现代意识，掌握现代知识，体现现代风貌。进一步解放思想，增强创新意识，打破习惯思维和做法，在实践中不断创新工作思路，改进工作方式方法，娴熟运用经济、法律、行政手段以及顺应和利用市场力量，提高精细化管理水平，促进和推动工作更有效率、更具成效。进一步发挥市长热线、市政府公众信息网和“12345”效能监察热线的作用，加强对群众所反映问题的受理与落实解决，在政府与群众之间搭建更便捷有效的沟通服务平台。进一步增强各级各部门领导干部为加快鹤壁发展不计得失、忘我工作、真抓实干、奋发进取的压力感、责任感和使命感，真心实意为群众办实事，刻苦努力为经济社会发展做贡献。加强公务员队伍培训与建设。围绕全市重点工作优化配置人力资源，把更多的力量充实到重要部门和关键岗位。充分运用电子政务和直接巡查暗访等手段，强化工作督查与监督，严格执行问责制，提高政府执行力，保证工作不折不扣地抓实抓好。认真落实廉政建设责任制，坚持廉洁从政、从严治政。

　　各位代表！回顾“十五”的成就，我们无比自豪；展望“十一五”前景，我们信心百倍。让我们在省委、省政府和市委的领导下，奋发有为，自强不息，推动我市经济社会跨越式发展，为实现“两个率先”、开创“十一五”经济社会发展新局面而努力奋斗！
